# Efficacy and Dosage Pattern of Sacubitril/Valsartan in Chinese Heart Failure with Reduced Ejection Fraction Patients

**DOI:** 10.1007/s12265-022-10236-x

**Published:** 2022-05-03

**Authors:** Iokfai Cheang, Shi Shi, Xinyi Lu, Shengen Liao, Xu Zhu, Xi Su, Qi Lu, Jing Yuan, Dachun Xu, Min Zhang, Cuilian Dai, Jingfeng Wang, Fang Yuan, Yan Zhao, Jingmin Zhou, Xinli Li

**Affiliations:** 1 Department of Cardiology, The First Affiliated Hospital of Nanjing Medical University, Guangzhou Road 300, Nanjing, 210029 People’s Republic of China; 2 Department of Cardiology, Wuhan Asia Heart Hospital, Wuhan, 430022 People’s Republic of China; 3 Department of Cardiology, Affiliated Hospital of Nantong University, Nantong, 226001 People’s Republic of China; 4 Department of Cardiology, Tongji Medical College of Huazhong University Affiliated Union Hospital, Wuhan, 430022 People’s Republic of China; 5 Department of Cardiology, Shanghai Tenth People’s Hospital, Tongji University School of Medicine, Shanghai, 200072 People’s Republic of China; 6 Department of Cardiology, Shijiazhuang Greatwall Hospital, Hebei, 052260 People’s Republic of China; 7 Department of Cardiology, Xiamen Cardiovascular Hospital, Xiamen University, Xiamen, 361010 People’s Republic of China; 8 Department of Cardiology, Sun Yat-Sen Memorial Hospital, Sun Yat-Sen University, Guangzhou, 510120 People’s Republic of China; 9 Department of Cardiology, Fuwai Central China Cardiovascular Hospital, Zhengzhou, 450008 People’s Republic of China; 10 Department of Cardiology, Xuzhou Central Hospital, Xuzhou, 221009 People’s Republic of China; 11 Department of Cardiology, Shanghai Institute of Cardiovascular Diseases, Zhongshan Hospital, Fudan University, 180 Fenglin Road, Shanghai, 200032 People’s Republic of China

**Keywords:** Angiotensin receptor-neprilysin inhibitor (ARNI), Heart failure, Sacubitril/valsartan, Chart review, Real-world data

## Abstract

This study aims to investigate the dosage pattern, efficacy, and safety of sacubitril/valsartan (Sac/Val) in Chinese heart failure with reduced ejection fraction (HFrEF) patients regarding real-world settings. Patients from 27 centers with a confirmed diagnosis of HFrEF and initiated Sac/Val treatment were enrolled. The primary objective was to evaluate the dosage pattern and change of heart failure status. In a final cohort of 983 patients, outpatient Sac/Val treatment demonstrated a similar beneficial effect in NT-proBNP and cardiac function. After initiating the treatment, overall and sub-population showed similar safety and efficacy. Patients who received a higher dose of Sac/Val (> 200 mg/d) demonstrated better improvement in LV function and reduction of NT-proBNP regardless of adjustment. Among Chinese HFrEF patients, Sac/Val showed a comparable reduction in NT-proBNP and improvement in cardiac function. Data further support guideline recommendations of Sac/Val in Chinese population. Optimal up-titration might provide further benefits. Further long-term and prognostic studies are needed.

**Graphical abstract:**

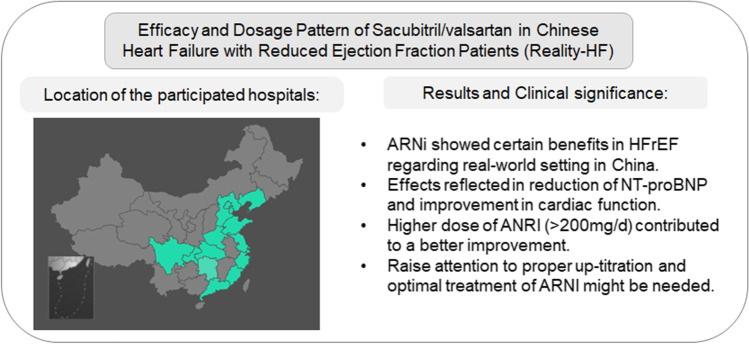

## Introduction

With the population aging and rapid urbanization in China, there are over 300 million people with cardiovascular disease (CVD) [1, 2]. As the end-stage syndrome of various CVD, the growing morbidity of heart failure (HF) increases health burden. In the past decade, numbers of novel pharmacological treatments have been shown to benefit HF patients [3].

Sacubitril/valsartan (Sac/Val), an angiotensin receptor neprilysin inhibitor (ARNI), significantly reduced cardiovascular mortality and HF hospitalizations as compared to angiotensin-converting enzyme inhibitors (ACEI) for patients with heart failure with reduced ejection fraction (HFrEF; LVEF < 40%) [4], was approved in July 2017 in China. Although PARADIGM-HF has a relatively low representation of Asian population, ARNI has been recommended for most HFrEF patients in Chinese and other global HF guidelines [5–7]. The growing evidence regarding the safety and efficacy of ARNI in improving HF prognosis [9–12] demonstrated reduction in NT-proBNP concentration and improvement in echocardiographic markers of cardiac function. [13] Previous studies also demonstrated an early treatment strategy for initiating time, up-titrating, and optimizing dose of ARNI in both hospitalized and outpatient settings could improve clinical outcomes in HFrEF [14–17]. With years of utilization, various data based on the guideline-directed medical therapy (GDMT) showed ARNI initiations were associated with early improvements in patient-reported health status [17, 18]. Furthermore, subgroup analyses regarding different comorbidities also showed additional evidence regarding its safety and effectiveness (e.g., PARADIGM-HF LFT, HRQL) [19–21].

However, the evidence of the titration patterns, prescribed dose, and patient characteristics in real-world clinical practice in the Chinese population were still missing, which limits the translation of conclusions to broad patient populations and the clinical implementation. While the sub-optimal use of ARNI in clinical practice remains high [8], such information is critical to inform physicians on translating the trial results into clinical practice for optimal usage of Sac/Val, especially in routine initiation and dose up-titration.

To better understand the treatment patterns of ARNI and improve clinical management for patients with HFrEF in real-world Chinese setting, this multi-center, retrospective, non-interventional, chart review study—Reality-HF—aims to provide clear dosage pattern, change of heart failure status, and safety report in HFrEF patients who were treated with Sac/Val.

## Methods

### Study Setting and Population

The study population was composed of HFrEF patients who started to receive Sac/Val from September 2017 to August 2018, at 27 tertiary hospitals in China (Appendix Table 1—list of participating hospitals). Patients were anonymized using a 9-digit number consisting of a 4-digit center number and a 5-digit patient number. The chart review, for HFrEF patients who were prescribed Sac/Val in the identification period (19 September 2017 to 30 August 2018), was extracted from the time of first Sac/Val prescription (index date), 6 months before (pre-index period), and after (post-index period) the index date for the baseline and clinical characteristics of patients (Appendix Fig. 1—study flowchart).

Medical charts of patients with the following characteristics were for data abstraction: (1) 18 years of age or older; (2) confirmed diagnosis of HFrEF by investigator; (3) patients prescribed Sac/Val during the identification period; (4) patient has at least 1 follow-up visit after index date. Patients who participated in other clinical study and confirmed pregnancy were excluded. To be noted, for the diagnosis of HFrEF, patients were first identified by the International Classification of Diseases-10: I50 (heart failure), and then checked by the results of echocardiogram for reduced left ventricular ejection fraction (LVEF).

To describe the study population, characteristics of demographics, comorbidities (anemia, hypertension (HTN), diabetes mellitus (DM), chronic kidney disease (CKD), chronic obstructive pulmonary disease), heart failure history, and physical examination were collected. Information on Sac/Val dosage, changes of heart failure status, and the parameters of comorbidities were also collected through the available charts. The 50 mg of Sac/Val contains 24 mg of sacubitril and 26 mg of valsartan. The total dosages in daily use were documented and further analyzed (mg/d).

This study protocol was approved by the independent Ethics Committee of the First Affiliated Hospital of Nanjing Medical University and China National Center for Biotechnology Development (GH0402).

### Primary and Secondary Objectives

The primary objective was to evaluate the dosage changes (e.g., increased dosage—up-titrated) in HFrEF patients receiving Sac/Val and their heart failure status based on functional and biomarker tests. Dosage at the index date to month 6 post-indexing date and its titration change were documented. Simultaneously, the New York Heart Association (NYHA) classification and NT-proBNP levels from baseline to months 1, 2, 3, 4, 5, and 6 post-indexing date in HFrEF patients receiving Sac/Val were documented.

Secondary objectives were to describe the treatment patterns and clinical status of HFrEF patients receiving Sac/Val over time. Exploratory secondary objectives also included the subgroup analysis of the Sac/Val dosage pattern in HFrEF patients by their comorbidities (HTN, DM, CKD) and corresponding parameters—systolic blood pressure (SBP) and diastolic blood pressure (DBP), serum creatinine, serum potassium, and echocardiographic parameters.

### Data Management

After the trained site investigators reviewed charts from hospital databases based on the inclusion/exclusion criteria, medical records of the eligible patients were extracted and anonymized. Admission information, drug administration record, diagnostic and treatment procedure records, laboratory results, physician notes, discharge summary, etc. collected using a web-based electronic case report form (eCRF) were encrypted and uploaded to a secured server ensuring confidentiality and privacy of individuals. Data from all participating hospitals were then combined for further analysis.

### Statistical methods

Independent statistical specialists at ClinChoice Inc. adopted PASS®11 and SAS®9.4 software (or higher version) package to the following statistical analysis. All tests were 2-sided, and *P* < 0.05 were considered statistically significant.

Missing values were not replaced. If prescription of Sac/Val or other drug classes were discontinued permanently, then the endpoints would be considered missing. Counts of missing values for both continuous and categorical variable were calculated. Descriptive data analyses were carried out to describe patient profiles, dosage pattern of Sac/Val, treatment patterns, and clinical parameters.

Continuous variables are presented as the means (standard deviations, SDs) with normal distribution or medians with interquartile ranges (IQRs) with non-normal distribution. Comparison was performed using the unpaired Student’s *t*-test or the Mann–Whitney *U*-test based on their distribution. Categorical variables are presented as numbers (%) and were compared using the Pearson *χ*^2^ test.

The assessment of differences in changes in NT-proBNP levels was based on analysis of covariance (ANCOVA) model. The response variable is the change from baseline in NT-proBNP level at each follow-up time point; age, sex, body mass index (BMI), SBP, creatinine, previous ACEI/angiotensin II receptor blockers (ARB) treatment, follow-up time points, and interaction between follow-up time points, and different target dose groups as fixed effects; and logarithm of baseline NT-proBNP as covariate.

## Results

### Baseline

The detailed baseline characteristics are shown in Table 1. In total, 983 patients were eligible for this study. The mean age was 57.3 years, and 80.5% patients were male. There were 46.2% of patients who had a history of HTN; 27.2% patients had history of DM; 12.4% patients had CKD; 38.8% of patients had a history of coronary heart disease (CHD); and 17.5% of patients had a history of myocardial infarction. ACEI/ARB were prescribed in 59.2% of patients prior to the Sac/Val treatment. Beta-blockers were prescribed in 66.6% of patients, and mineralocorticoid receptor antagonists (MRA) were prescribed in 63.9% of patients. The N-terminal pro B-type natriuretic peptide (NT-proBNP) level at baseline was 2480.61 pg/mL.

**Table 1:** Baseline characteristic

Variable	Valid *N*	Baseline
Age, year	983	57.3 (14.81)
Male	983	791 (80.5%)
Han ethnicity	983	971 (98.9%)
BMI, kg/m^2^	706	24.62 (4.221)
Systolic blood pressure, mmHg	931	115.9 (19.30)
Diastolic blood pressure, mmHg	931	73.0 (13.32)
LVEF, %	820	29.94 (6.701)
NT-proBNP, pg/mL	661	2480.61 (1005.00–5652.00)
Serum creatinine, μmol/L	859	98.704 (54.824)
Potassium, mmol/L	890	4.100 (0.467)
Medical history	983	
Hypertension		454 (46.2%)
Diabetes mellitus		267 (27.2%)
Chronic kidney disease		122 (12.4%)
Arrhythmia		320 (32.5%)
Dilated cardiomyopathy		496 (50.5%)
Coronary heart disease		381 (38.8%)
Myocardial infarction		172 (17.5%)
Medication	983	
ACEI/ARB		583 (59.2%)
Diuretics		663 (67.4%)
Beta-blocker		655 (66.6%)
MRA		628 (63.9%)
None		89 (9.1%)
NYHA functional class	778	
II		141 (17.9%)
III		368 (46.7%)
IV		269 (34.1%)
Highest dose during follow-up	983	
< 200 mg/d		643 (65.4%)
≥ 200 mg/d		340 (34.6%)

Data are presented as mean (SD), median (25th, 75th percentile) or *n* (%)

*LVEF* left ventricular ejection fraction; *NT-proBNP* N-terminal pro B-type natriuretic peptide; *ACEI* angiotensin-converting enzyme inhibitor; *ARB* angiotensin-receptor blocker; *MRA* mineralocorticoid receptor antagonists

### Primary Objective

#### Dosage Patterns and Utilization of Sac/Val

**Fig. 1:**
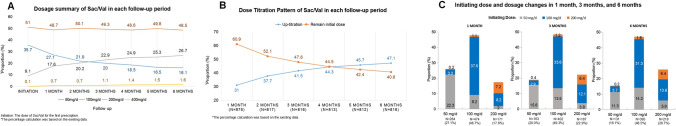
Dosage pattern. **A** Dosage summary of Sac/Val in each follow-up period. **B** Dose titration pattern of Sac/Val in each follow-up period. **C** Initiating dose and dosage changes in 1 month, 3 months, and 6 months. Initiation: The dose of Sac/Val for the first prescription. *The percentage calculation was based on the existing data

There were 35.7% and 51% of patients who initiated Sac/Val in ≤ 50 mg/d and 100 mg/d, respectively (Fig. 1A). Compared with the initial dose, 56.7% of patients had a dose change during the follow-up period. During the 6-month follow-up, there were 291 (35.7%) patients up-titrated steadily. The proportion of patients who up-titrated showed an upward trend (Fig. 1B), and patients who received ≥ 200 mg dose up-titrated from the initial dose of ≤ 50 and 100 mg gradually increased (Fig. 1C).

#### Change in Plasma NT-proBNP Level and NYHA Functional Classification

**Fig. 2:**
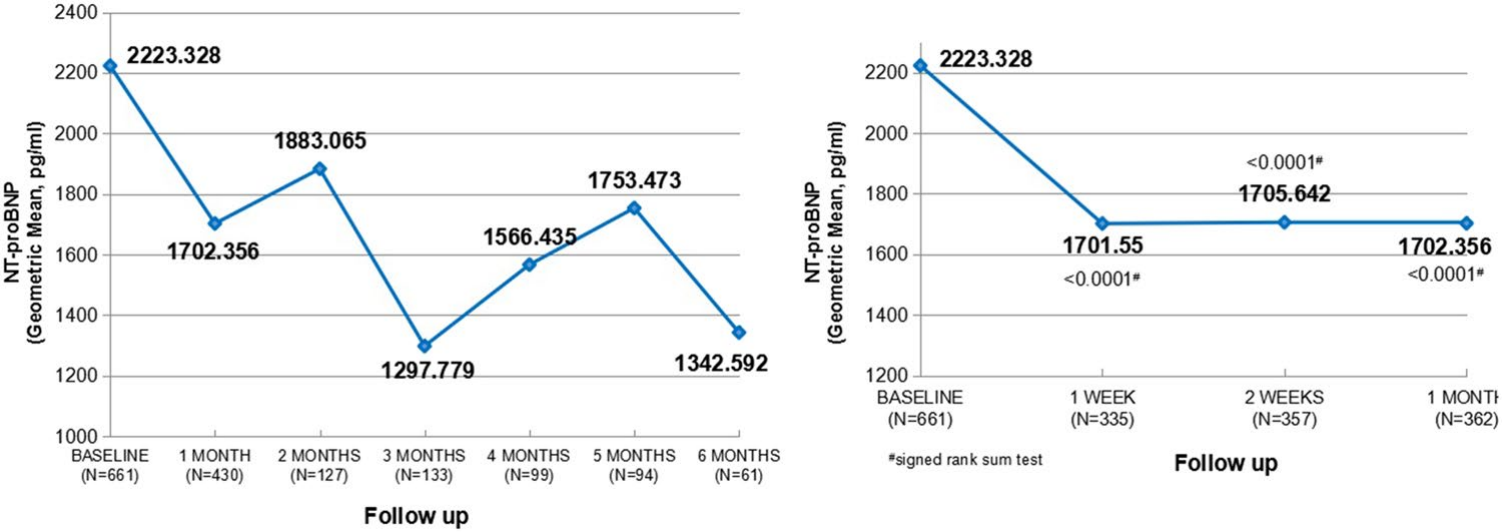
Changes of NT-proBNP levels in HFrEF patients receiving Sac/Val

During the 6 months of treatment, a favorable effect was observed on the plasma NT-proBNP level from baseline after initiating Sac/Val. Although the changes did not show significance in every time-point, NT-proBNP were significantly decreased especially in the early stage of treatment (1 week, 2 weeks, and 1 month vs baseline, all *P* < 0.0001, Fig. 2).

In a model further adjusted with age, sex, BMI, SBP, and creatinine (Table 2), the difference of log-transferred NT-proBNP level in the follow-up time-points remained statistically significant compared to baseline (1 month vs baseline, OR = 1.304, 95% CI (1.019, 1.670), *P* = 0.0351; 3 months vs baseline, OR = 2.343, 95% CI (1.616, 3.398), *P* < 0.0001; 6 months vs baseline, OR = 2.587, 95% CI (1.381, 4.848), *P* = 0.0031).

**Table 2:** Difference in log-transferred NT-proBNP compared to baseline

Follow-up time	Available *N*	< 200 mg/d, 95% CI	≥ 200 mg/d, 95% CI	OR	*P*
1 months	861	0.601 (0.520, 0.694)	0.460 (0.377, 0.563)	1.304 (1.019, 1.670)	0.0351
3 months	430	0.733 (0.577, 0.930)	0.313 (0.235, 0.416)	2.343 (1.616, 3.398)	< 0.0001
6 months	133	0.759 (0.531, 1.084)	0.293 (0.175, 0.492)	2.587 (1.381, 4.848)	0.0031

Model adjusted for age, sex, body mass index, SBP, creatinine, previous ACEI/ARB treatment, different target dose groups, follow-up time points, and interaction between follow-up time points as fixed effects

*95% CI* 95% confidence interval; *OR* odd ratio

Patients’ cardiac function also showed a favorable effect which is represented in the improvement of NYHA functional classification (Appendix Fig. 2—improvement of NYHA classification in HFrEF patients receiving Sac/Val**)**.

### Secondary Objective

#### Echocardiographic Parameters

Table 3 demonstrates the results of echocardiographic parameters with each follow-up time-point. Compared with baseline, patients with HFrEF after 6 months of treatment with Sac/Val have showed varying degrees of improvement in the echocardiographic parameters.

**Table 3:** Echocardiogram measurements

Parameter	Baseline	1 month	2 months	3 months	4 months	5 months	6 months
LVEF (%)	**820**	**381**	**143**	**166**	**127**	**112**	**92**
Median	29.94	31.3	36.0	36.0	36.0	33.5	37.5
Change from baseline (Median)		2.8	4	6	5	4	6
Mean (SD)	29.96 (6.72)	32.82 (9.51)	35.95 (9.53)	37.13 (11.15)	36.31 (12.38)	35.87 (13.30)	38.39 (11.44)
Change from baseline (Mean)		4.14	6.49	7.82	6.81	7.04	8.63
*P value		< 0.0001	< 0.0001	< 0.0001	< 0.0001	< 0.0001	< 0.0001
LVDd (mm)	**834**	**381**	**143**	**166**	**127**	**112**	**92**
Median	66.0	66.0	65.0	61.0	64.0	65.0	64.0
Change from baseline (Median)		− 1	− 1	− 2	− 2	− 1	− 2
Mean (SD)	66.54 (10.03)	66.50 (11.88)	65.70 (10.44)	63.94 (10.58)	65.82 (17.95)	66.26 (13.92)	63.98 (9.25)
Change from baseline (Mean)		− 1.55	− 2.20	− 3.29	− 1.70	− 2.64	− 3.69
*P value		< 0.0001	< 0.0001	< 0.0001	< 0.0001	0.0044	< 0.0001
LAD (mm)	**765**	**368**	**137**	**157**	**124**	**110**	**89**
Median	47.0	45.0	46.0	44.0	44.5	45.0	43.0
Change from baseline (Median)		− 2	− 2	− 3	− 2	− 2	− 3
Mean (SD)	47.75 (8.09)	45.57 (8.70)	46.55 (7.90)	44.87 (7.74)	44.49 (7.97)	46.70 (8.96)	44.78 (6.51)
Change from baseline (Mean)		− 1.85	− 2.44	− 3.74	− 3.35	− 2.26	− 3.17
*P value		< 0.0001	< 0.0001	< 0.0001	< 0.0001	0.0114	< 0.0001

*LVEF* left ventricle ejection fraction; *LVDd* left ventricle diastolic diameter; *LAD* left atrial diameter; *SD* standard deviations. *Paired *T*-test

Mean LVEF was 29.96 (6.72) % in baseline, and the LVEF at each follow-up time-point after baseline showed varying increase (1 month, 32.82 (9.51), *P* < 0.0001; 3 months, 37.13 (11.15), *P* < 0001).

Mean Left ventricle diastolic diameter (LVDd) at baseline was 66.54 (10.03) mm, and the LVDd at each follow-up time-point decreased significantly from baseline (1 month, 66.50 (11.88), *P* < 0.0001; 3 months, 63.94 (10.58), *P* < 0.0001).

Mean left atrial diameter (LAD) at baseline was 47.75 (8.09) mm, and the LAD at each follow-up time-point of left atrial diameter (LAD) also decreased significantly from baseline (1 month, 45.57 (8.70), *P* < 0.0001; 3 months, 44.87 (7.74), *P* < 0.0001).

#### Safety Analysis

During 6 months of treatment with Sac/Val, 21 (2.1%) patients had increased serum creatinine (≥ 3.5 mg/dl / 309.4 umol/L); 43 (4.4%) patients had increased serum potassium (≥ 5.5 mmol/L); and 26 (2.6%) patients complained episode of symptomatic hypotensive events.

Most of the increased creatinine events occurred among the CKD patients (15 out of 122, 12.3%, *P* < 0.001). Occurrence of increased potassium were also significantly higher in CKD patients (13 out of 122, 10.7%, *P* = 0.003).

#### Re-hospitalization Rate

There were 743 (75.6%) patients who had at least one hospitalization event 6 months prior to the index date. After initiation of Sac/Val, there were 368 (37.4%) patients who were hospitalized during follow-up. Regardless of the baseline dose and titration mode, the hospitalization rate of patients showed a downward trend (Appendix Fig. 3—hospitalization at each follow-up).

**Fig. 3:**
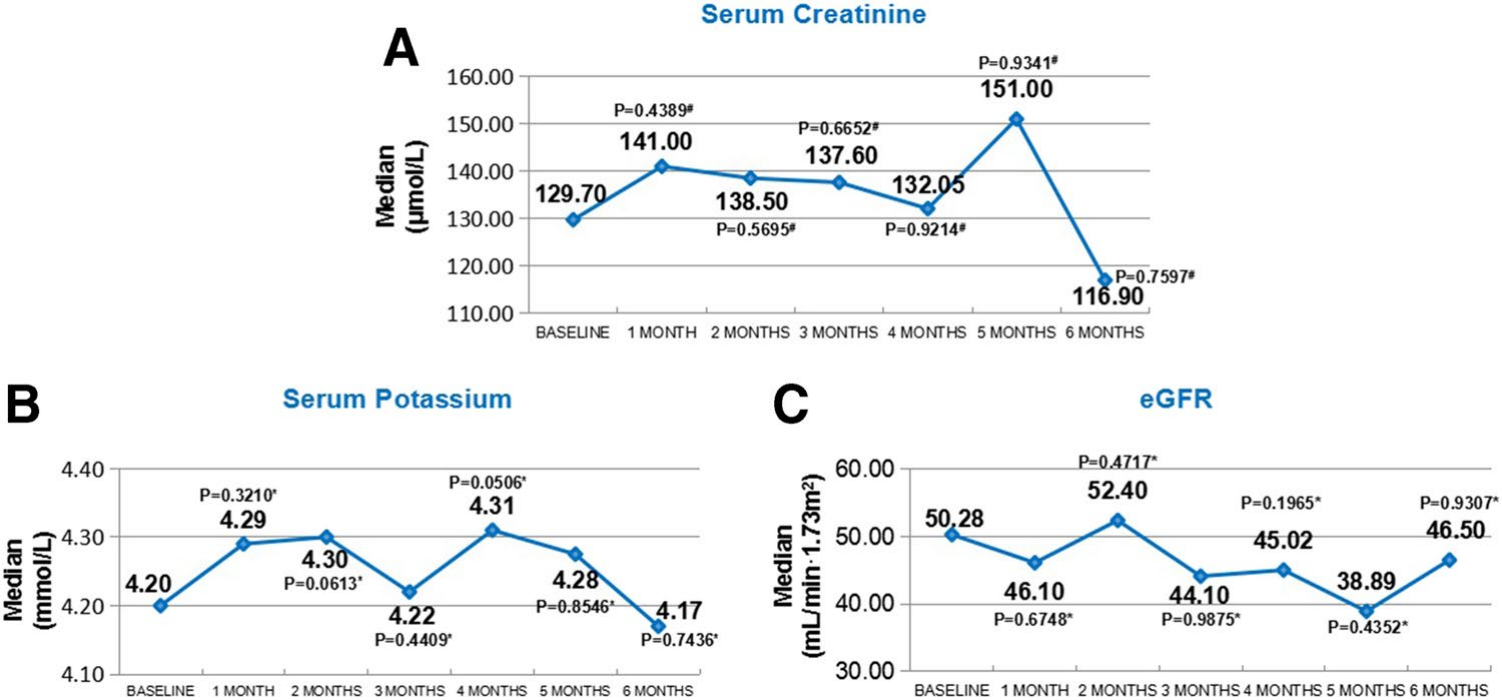
Change of renal function and potassium in HFrEF patients with chronic kidney disease receiving Sac/Val. **A** Serum creatinine; **B** serum potassium; **C** estimated glomerular filtration rate. *paired *T* test, #signed rank sum test

#### Subgroup Analysis

In the subgroup analyses with different medical histories, comparing the patients with/without previous AECI/ARB treatment or comorbidities (e.g., HTN, DM, CKD, CHD, DCM), there were no significant difference in the 6-month reduction of NT-proBNP and the improvement of LVEF (Appendix Fig. 4—subgroup analysis of comorbidity).

**Fig. 4:**
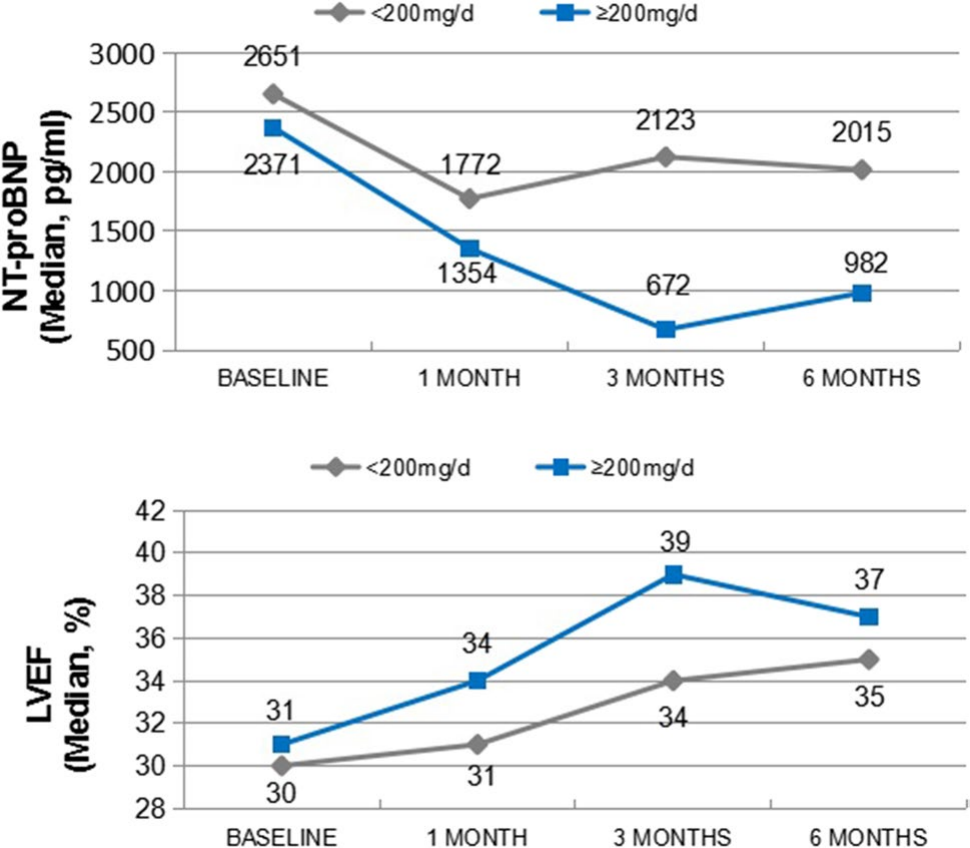
Characteristics and cardiac improvement in different dosage group

Although the increased creatinine events mainly occurred in the subgroup of CKD patients, results showed the median of serum creatinine, potassium, and estimated glomerular filtration rate remained stable during the Sac/Val treatment compared to baseline (All *P* > 0.05, Fig. 3).

Also, compared to baseline, patients who received a higher dose of ARNI (≥ 200 mg/d) showed a significant reduction in NT-proBNP (*P* = 0.0351) and improvement of cardiac function (*P* = 0.0076) in follow-up (Table 4, Fig. 4).

**Table 4:** Cardiac improvement in different dosage group

Characteristics	< 200 mg/d (*N* = 644)	≥ 200 mg/d (*N* = 340)	*P*
Age, Mean (SD)	57.8 (14.64)	56.2 (15.07)	0.150
Male, *n* (%)	508 (79.0%)	284 (83.5%)	0.086
BMI (kg/m2)			0.0012
Valid N	490	216	
Mean (SD)	24.24 (4.114)	25.46 (4.347)	
Baseline LVEF (%)			0.0076
Valid N	552	268	
Mean (SD)	29.77 (6.943)	30.29 (6.171)	
Follow-up LVEF (%)			
Valid N	253	171	
Mean (SD)	35.38 (11.017)	39.85 (10.887)	
Baseline NT-proBNP(pg/mL)			0.0351
Valid N	442	219	
Mean value	2650.90	2371.00	
Follow-up NT-proBNP (pg/mL)			
Valid N	273	157	
Mean value	2015.00	982.00	

## Discussion

To the best of our knowledge, the current study firstly reviewed the efficacy of ARNI in HFrEF patients regarding real-world setting in Mainland China. The effects reflected in reduction of NT-proBNP and improvement in cardiac function. A higher dose of ARNI (> 200 mg/d) might contribute to a better improvement. These results further support the generalization of the previous RCT results in Chinese population.

Despite the various evidence regarding ARNI treatment in HF, most of these RCTs and registered studies were conducted with certain trial criteria and/or among the predominantly non-Hispanic White populations, which limit the results generalized into other ethnic subgroups [22–24]. Also, the adverse effects such as renal dysfunction and hyperkalemia, hypotension, angioedema, and other systemic impact for racial and ethnic differences were the other main areas of concern. While Asian patients with HF are often younger and have worse outcomes than the global average [25–27], it has also been suggested that the ethnic differences [28–30] in tolerability, efficacy, and safety of cardiovascular drugs may be derived from the physical characteristics, pharmacokinetics, innate genetic polymorphisms, lifestyle, etc. Meanwhile, these ethnic and population differences in salt sensitivity and natriuretic peptide–mediated mechanism [21, 31] might also cause a distinct pharmacological effect in treating heart failure for Chinese population.

Understanding the ethnic differences in heart failure treatment will help us develop more personalized diagnosis and could optimize patient management. In previous analysis focused on Taiwan region [32], the composing proportion of comorbidities and medications in baseline was different from our study; and the study only included BNP as the heart failure biomarker which is known to be affected by neprilysin inhibitor [33, 34]. In addition, similar Asian population of Japanese HF patient with renal dysfunction showed ARNI was a promising option to preserve the renal function and improve clinical outcomes [35, 36], but the small-scale and short follow-up impeded the generalization. Therefore, our real-world data from Mainland China provided a certain verification of the effectiveness and safety.

Regarding the dosage and titration aspect, the initiating time, up-titrating, and optimizing dose of ARNI contribute to the improvement in HF prognosis [8, 37]. In a Japanese population, Sac/Val therapy initiated at a lower dose was safe and may be effective in Japanese heart failure patients [38]. In the present study, we observed that patients often initiated with sacubitril/valsartan at a relatively lower dose have difficulty in up-titrating the dose due to the concerns of orthostatic hypotension. After initiating treatment with Sac/Val, the current dosage patterns in both overall and sub-population of different comorbidities were safe and well-tolerated, similar to the previous results. Therefore, we are emphasizing the importance of target dose optimization and GDMT where proper medical and social supports are needed in clinical practice.

NT-proBNP as the benchmark heart failure biomarker offers prognostic information, independent of standard clinical predictors, and refines risk stratification. Although reference interval could be affected by various factors, the overall diagnostic performance of NT-proBNP remained in different population [39, 40]. As shown in these studies [4, 8, 11, 41] and more, treatment with ARNI has significant reductions in NT-proBNP especially observed as early as 2 weeks which were sustained throughout the treatment period. Our result demonstrated a reduction of NT-proBNP in the early stage after initiation which consisted with the previous studies, suggesting that Chinese patients could derive similar benefit from Sac/Val in improving NT-proBNP and cardiac function.

Also, patients who received a higher dose in the study showed a better improvement. Compared with the patients who failed to up-titrate during outpatient follow-up, patients who received a higher dose of ARNI (> 200 mg/d) demonstrated a better improvement in LV function and reduction of NT-proBNP. The improvement of NT-proBNP remained significantly higher in > 200 mg/d group in the adjusted model which further supports the significance to improve this sub-optimal treatment pattern in Mainland China.

In real-world clinical practice, up-titration of ARNI remains challenging due to inherent risks of these untoward adverse effects such as symptomatic hypotension, kidney function impairment, and/or hyperkalemia. Nonetheless, previous studies showed Sac/Val led to a lower incidence of renal deterioration compared with ACEI or ARB alone [42, 43]. Similar beneficial effects in patients with CKD on kidney function and albuminuria [44, 45]. In the safety and subgroup analysis of the current study, the benefits of Sac/Val were not modified by the comorbidity. Similar safety and improvement trend in keeping with prior reports were found in our result, suggesting the previous evidence could be generalized to Chinese HFrEF patients. In selected patients, especially CKD patients, closer follow-ups are needed for monitoring the possible adverse reaction when initiated within outpatient settings.

To our knowledge, this study represents the first analysis providing the initiation of the ARNI treatment patterns in China. Our data suggests that the up-titration of ARNI remains sub-optimal in real-world clinical practice in Mainland China. On the other hand, treatment with ARNI under the current dosage was safe even in patients with CKD. Improved recognition in primary health care and titration management of ARNI are needed for advance up-titration and optimal treatment.

## Limitation

Our study represents the first real-world evidence of the ARNI dosage pattern and clinical efficacy in Chinese HFrEF patients. However, several limitations exist for our analysis, as this is a retrospective, observational study. Incomplete data and lack of internal validity in certain aspect might have led to bias and/or limited the generalization. Secondly, a relatively short follow-up limited the assumption of further outcome of all-cause mortality and treatment effects. Thirdly, safety reporting was only done on comorbidity and an aggregate level; the long-term survival details of each patient were not available. Lastly, the improvement of re-hospitalization might be the effect with standard heart treatment and not be solely driven by Sac/Val. A prospective cohort study with longer follow-up period could further eliminate the bias of these factors.

## Conclusion

In this retrospective real-world analysis, sacubitril/valsartan demonstrated a similar efficacy and safety in Mainland Chinese HFrEF patients, which is characterized by the NT-proBNP reduction and cardiac function improvement. The current dosage pattern in Mainland China remained sub-optimal, and proper up-titration of ARNI and enhanced medical management might be needed.

## Data Availability

Derived data of this study are available from the corresponding author XL. L on reasonable request.

## References

[1] The Writing Committee of the Report on Cardiovascular Health and Diseases in China (2019) Report on Cardiovascular Health and Diseases in China 2019: An Updated Summary. *Chinese Circulation Journal,* *35*(9): 833–854. 10.3969/j.issn.1000-3614.2020.09.001

[2] Wang H, Chai K, Du M, Wang S, Cai JP, Li Y, Zeng P, Zhu W, Zhan S, Yang J (2021) Prevalence and incidence of heart failure among urban patients in china: A national population-based analysis. *Circulation: Heart Failure,**14*(10):e008406. 10.1161/CIRCHEARTFAILURE.121.008406. Epub 2021 Aug 28. PMID: 34455858. 10.1161/CIRCHEARTFAILURE.121.008406 34455858

[3] Crespo-Leiro MG, Metra M, Lund LH, Milicic D, Costanzo MR, Filippatos G, Gustafsson F, Tsui S, Barge-Caballero E, De Jonge N, Frigerio M, Hamdan R, Hasin T, Hülsmann M, Nalbantgil S, Potena L, Bauersachs J, Gkouziouta A, Ruhparwar A, Ristic AD, Straburzynska-Migaj E, McDonagh T, Seferovic P, Ruschitzka F (2018). Advanced heart failure: A position statement of the Heart Failure Association of the European Society of Cardiology. European Journal of Heart Failure.

[4] McMurray JJ, Packer M, Desai AS, Gong J, Lefkowitz MP, Rizkala AR, Rouleau JL, Shi VC, Solomon SD, Swedberg K, Zile MR (2014) PARADIGM-HF Investigators and Committees. Angiotensin-neprilysin inhibition versus enalapril in heart failure. *The New English Journal of Medicine, 371*(11):993–1004. 10.1056/NEJMoa1409077. Epub 2014 Aug 30. PMID: 25176015. 10.1056/NEJMoa1409077 25176015

[5] Heart Failure Group of Chinese Society of Cardiology of Chinese Medical Association; Chinese Heart Failure Association of Chinese Medical Doctor Association; Editorial Board of Chinese Journal of Cardiology. [Chinese guidelines for the diagnosis and treatment of heart failure 2018]. Zhonghua Xin Xue Guan Bing Za Zhi. 2018 Oct 24;46(10):760–789. Chinese. 10.3760/cma.j.issn.0253-3758.2018.10.004. PMID: 30369168. 10.3760/cma.j.issn.0253-3758.2018.10.004 30369168

[6] Yancy CW, Jessup M, Bozkurt B, Butler J, Casey DE, Colvin MM, Drazner MH, Filippatos GS, Fonarow GC, Givertz MM, Hollenberg SM, Lindenfeld J, Masoudi FA, McBride PE, Peterson PN, Stevenson LW, Westlake C (2017). 2017 ACC/AHA/HFSA Focused Update of the 2013 ACCF/AHA Guideline for the Management of Heart Failure: A report of the American College of Cardiology/American Heart Association Task Force on Clinical Practice Guidelines and the Heart Failure Society of America. Circulation.

[7] Seferovic PM, Ponikowski P, Anker SD, Bauersachs J, Chioncel O, Cleland JGF, de Boer RA, Drexel H, Ben Gal T, Hill L, Jaarsma T, Jankowska EA, Anker MS, Lainscak M, Lewis BS, McDonagh T, Metra M, Milicic D, Mullens W, Piepoli MF, Rosano G, Ruschitzka F, Volterrani M, Voors AA, Filippatos G, Coats AJS (2019) Clinical practice update on heart failure 2019: pharmacotherapy, procedures, devices and patient management. An expert consensus meeting report of the Heart Failure Association of the European Society of Cardiology. *European Journal of Heart Failure, 21*(10):1169–1186. 10.1002/ejhf.1531. Epub 2019 Aug 30. PMID: 31129923. 10.1002/ejhf.1531 31129923

[8] Greene SJ, Butler J, Albert NM, DeVore AD, Sharma PP, Duffy CI, Hill CL, McCague K, Mi X, Patterson JH, Spertus JA, Thomas L, Williams FB, Hernandez AF, Fonarow GC (2018). Medical Therapy for Heart Failure With Reduced Ejection Fraction: The CHAMP-HF Registry. Journal of the American College of Cardiology.

[9] Sauer AJ, Cole R, Jensen BC (2019). Practical guidance on the use of sacubitril/valsartan for heart failure. Heart Failure Reviews.

[10] Solomon SD, Vaduganathan M, L Claggett B, Packer M, Zile M, Swedberg K, Rouleau J, A Pfeffer M, Desai A, Lund LH, Kober L, Anand I, Sweitzer N, Linssen G, Merkely B, Luis Arango J, Vinereanu D, Chen CH, Senni M, Sibulo A, Boytsov S, Shi V, Rizkala A, Lefkowitz M, McMurray JJV (2020) Sacubitril/Valsartan Across the Spectrum of Ejection Fraction in Heart Failure. *Circulation*, *141*(5):352–361. 10.1161/CIRCULATIONAHA.119.044586. Epub 2019 Nov 17. PMID: 31736342. 10.1161/CIRCULATIONAHA.119.044586 31736342

[11] Januzzi JL Jr, Prescott MF, Butler J, Felker GM, Maisel AS, McCague K, Camacho A, Piña IL, Rocha RA, Shah AM, Williamson KM, Solomon SD (2019) PROVE-HF Investigators. Association of Change in N-Terminal Pro-B-Type Natriuretic Peptide Following Initiation of Sacubitril-Valsartan Treatment With Cardiac Structure and Function in Patients With Heart Failure With Reduced Ejection Fraction. *JAMA 322*(11):1–11. 10.1001/jama.2019.12821. Epub ahead of print. PMID: 31475295; PMCID: PMC6724151. 10.1001/jama.2019.12821 PMC6724151 31475295

[12] Desai AS, Solomon SD, Shah AM, Claggett BL, Fang JC, Izzo J, McCague K, Abbas CA, Rocha R, Mitchell GF; EVALUATE-HF Investigators (2019) Effect of sacubitril-valsartan vs enalapril on aortic stiffness in patients with heart failure and reduced ejection fraction: A randomized clinical trial. *JAMA, 322*(11):1–10. 10.1001/jama.2019.12843. Epub ahead of print. PMID: 31475296; PMCID: PMC6749534. 10.1001/jama.2019.12843 PMC6749534 31475296

[13] Ibrahim NE, Piña IL, Camacho A, Bapat D, Felker GM, Maisel AS, Butler J, Prescott MF, Abbas CA, Solomon SD, Januzzi JL Jr; Prospective study of biomarkers, symptom improvement and ventricular remodeling during entresto therapy for heart failure (PROVE-HF) study investigators (2020) Racial and Ethnic Differences in Biomarkers, Health Status, and Cardiac Remodeling in Patients With Heart Failure With Reduced Ejection Fraction Treated With Sacubitril/Valsartan. *Circulation: Heart Failure*, *13*(11):e007829. 10.1161/CIRCHEARTFAILURE.120.007829. Epub 2020 Oct 3. PMID: 33016100. 10.1161/CIRCHEARTFAILURE.120.007829 PMC7769180 33016100

[14] Morrow DA, Velazquez EJ, DeVore AD, Desai AS, Duffy CI, Ambrosy AP, Gurmu Y, McCague K, Rocha R, Braunwald E (2019). Clinical outcomes in patients with acute decompensated heart failure randomly assigned to sacubitril/valsartan or enalapril in the PIONEER-HF Trial. Circulation.

[15] Senni M, McMurray JJ, Wachter R, McIntyre HF, Reyes A, Majercak I, Andreka P, Shehova-Yankova N, Anand I, Yilmaz MB, Gogia H, Martinez-Selles M, Fischer S, Zilahi Z, Cosmi F, Gelev V, Galve E, Gómez-Doblas JJ, Nociar J, Radomska M, Sokolova B, Volterrani M, Sarkar A, Reimund B, Chen F, Charney A (2016) Initiating sacubitril/valsartan (LCZ696) in heart failure: results of TITRATION, a double-blind, randomized comparison of two uptitration regimens.* European Journal of Heart Failure, 18*(9):1193–202. 10.1002/ejhf.548. Epub 2016 May 12. PMID: 27170530; PMCID: PMC5084812. 10.1002/ejhf.548 PMC5084812 27170530

[16] Wachter R, Senni M, Belohlavek J, Straburzynska-Migaj E, Witte KK, Kobalava Z, Fonseca C, Goncalvesova E, Cavusoglu Y, Fernandez A, Chaaban S, Bøhmer E, Pouleur AC, Mueller C, Tribouilloy C, Lonn E, A L Buraiki J, Gniot J, Mozheiko M, Lelonek M, Noè A, Schwende H, Bao W, Butylin D, Pascual-Figal D; TRANSITION Investigators (2019) Initiation of sacubitril/valsartan in haemodynamically stabilised heart failure patients in hospital or early after discharge: primary results of the randomised TRANSITION study. *European Journal of Heart Failure, 21*(8):998–1007. 10.1002/ejhf.1498. Epub 2019 May 27. PMID: 31134724.

[17] Packer M, McMurray JJ, Desai AS, Gong J, Lefkowitz MP, Rizkala AR, Rouleau JL, Shi VC, Solomon SD, Swedberg K, Zile M, Andersen K, Arango JL, Arnold JM, Bělohlávek J, Böhm M, Boytsov S, Burgess LJ, Cabrera W, Calvo C, Chen CH, Dukat A, Duarte YC, Erglis A, Fu M, Gomez E, Gonzàlez-Medina A, Hagège AA, Huang J, Katova T, Kiatchoosakun S, Kim KS, Kozan Ö, Llamas EB, Martinez F, Merkely B, Mendoza I, Mosterd A, Negrusz-Kawecka M, Peuhkurinen K, Ramires FJ, Refsgaard J, Rosenthal A, Senni M, Sibulo AS Jr, Silva-Cardoso J, Squire IB, Starling RC, Teerlink JR, Vanhaecke J, Vinereanu D, Wong RC; PARADIGM-HF Investigators and Coordinators 2015 Angiotensin receptor neprilysin inhibition compared with enalapril on the risk of clinical progression in surviving patients with heart failure. *Circulation,* *131*(1):54–61. 10.1161/CIRCULATIONAHA.114.013748. Epub 2014 Nov 17. PMID: 25403646. 10.1161/CIRCULATIONAHA.114.013748 25403646

[18] Luo N, Fonarow GC, Lippmann SJ, Mi X, Heidenreich PA, Yancy CW, Greiner MA, Hammill BG, Hardy NC, Turner SJ, Laskey WK, Curtis LH, Hernandez AF, Mentz RJ, O'Brien EC (2017). Early Adoption of Sacubitril/Valsartan for Patients With Heart Failure With Reduced Ejection Fraction: Insights From Get With the Guidelines-Heart Failure (GWTG-HF). JACC Heart Failure.

[19] Suzuki K, Claggett B, Minamisawa M, Packer M, Zile MR, Rouleau J, Swedberg K, Lefkowitz M, Shi V, McMurray JJV, Zucker SD, Solomon SD (2020). Liver function and prognosis, and influence of sacubitril/valsartan in patients with heart failure with reduced ejection fraction. European Journal of Heart Failure.

[20] Nielsen EE, Feinberg JB, Bu FL, Hecht Olsen M, Raymond I, Steensgaard-Hansen F, Jakobsen JC (2020). Beneficial and harmful effects of sacubitril/valsartan in patients with heart failure: A systematic review of randomised clinical trials with meta-analysis and trial sequential analysis. Open Heart.

[21] Berardi C, Braunwald E, Morrow DA, Mulder HS, Duffy CI, O’Brien TX, Ambrosy AP, Chakraborty H, Velazquez EJ, DeVore AD; PIONEER-HF Investigators (2020) Angiotensin-neprilysin inhibition in Black Americans: Data from the PIONEER-HF Trial. *JACC Heart Failure, 8*(10):859–866. 10.1016/j.jchf.2020.06.019. Epub 2020 Sep 9. PMID: 32919915. 10.1016/j.jchf.2020.06.019 32919915

[22] Correale M, Monaco I, Ferraretti A, Tricarico L, Padovano G, Formica ES, Tozzi V, Grazioli D, Di Biase M, Brunetti ND (2019). Hospitalization cost reduction with sacubitril-valsartan implementation in a cohort of patients from the Daunia Heart Failure Registry. International Journal of Cardiology Heart and Vasculature.

[23] Chng BLK, Hon JS, Chan H, Zheng Y, Gao F, Teo LYL, Sim KLD. Safety and tolerability of sacubitril/valsartan initiation in inpatient versus outpatient setting: A retrospective real world study. Heart, Lung and Circultaion 2020 Oct 5:S1443–9506(20)30449–2. 10.1016/j.hlc.2020.08.014. Epub ahead of print. PMID: 33032893.

[24] Wirtz HS, Sheer R, Honarpour N, Casebeer AW, Simmons JD, Kurtz CE, Pasquale MK, Globe G (2020) Real-world analysis of guideline-based therapy after hospitalization for heart failure. *Journal of the American Heart Association, 9*(16):e015042. 10.1161/JAHA.119.015042. Epub 2020 Aug 4. PMID: 32805181. 10.1161/JAHA.119.015042 PMC7660806 32805181

[25] Zhang Y, Zhang J, Butler J, Yang X, Xie P, Guo D, Wei T, Yu J, Wu Z, Gao Y, Han X, Zhang X, Wen S, Anker SD, Filippatos G, Fonarow GC, Gan T, Zhang R; China-HF Investigators (2014) Contemporary epidemiology, management, and outcomes of patients hospitalized for heart failure in China: Results from the China Heart Failure (China-HF) Registry. *Journal of Cardiac Failure*, 23(12):868–875. 10.1016/j.cardfail.2017.09.014. Epub 2017 Oct 10. PMID: 29029965. 10.1016/j.cardfail.2017.09.014 29029965

[26] Dokainish H, Teo K, Zhu J, Roy A, AlHabib KF, ElSayed A, Palileo-Villaneuva L, Lopez-Jaramillo P, Karaye K, Yusoff K, Orlandini A, Sliwa K, Mondo C, Lanas F, Prabhakaran D, Badr A, Elmaghawry M, Damasceno A, Tibazarwa K, Belley-Cote E, Balasubramanian K, Islam S, Yacoub MH, Huffman MD, Harkness K, Grinvalds A, McKelvie R, Bangdiwala SI, Yusuf S; INTER-CHF Investigators (2017) Global mortality variations in patients with heart failure: results from the International Congestive Heart Failure (INTER-CHF) prospective cohort study. *Lancet Glob Health, 5*(7):e665-e672. 10.1016/S2214-109X(17)30196-1. Epub 2017 May 3. Erratum in: The Lancet Global Health. 2017 Jul;5(7):e664. PMID: 28476564. 10.1016/S2214-109X(17)30196-1 28476564

[27] Bhopal RS, Bansal N, Fischbacher CM, Brown H, Capewell S; Scottish Health and Ethnicity Linkage Study (2012) Ethnic variations in heart failure: Scottish Health and Ethnicity Linkage Study (SHELS). *Heart, 98*(6):468–73. 10.1136/heartjnl-2011-301191. Epub 2012 Jan 27. PMID: 22285972. 10.1136/heartjnl-2011-301191 22285972

[28] Hasan MS, Basri HB, Hin LP, Stanslas J (2013). Genetic polymorphisms and drug interactions leading to clopidogrel resistance: Why the Asian population requires special attention. International Journal of Neuroscience.

[29] Dewan P, Docherty KF, McMurray JJV (2019). Sacubitril/valsartan in Asian patients with heart failure with reduced ejection fraction. Korean Circulation Journal.

[30] Gibson CM, Yuet WC (2021). Racial and ethnic differences in response to anticoagulation: A review of the literature. Journal of Pharmacy Practice.

[31] Huo Y, Li W, Webb R, Zhao L, Wang Q, Guo W (2019) Efficacy and safety of sacubitril/valsartan compared with olmesartan in Asian patients with essential hypertension: A randomized, double-blind, 8-week study. *Journal of Clinical Hypertension (Greenwich), 21*(1):67–76. 10.1111/jch.13437. Epub 2018 Dec 11. PMID: 30536595; PMCID: PMC8030324. 10.1111/jch.13437 PMC8030324 30536595

[32] Hsiao FC, Wang CL, Chang PC, Lu YY, Huang CY, Chu PH (2020). Angiotensin receptor neprilysin inhibitor for patients with heart failure and reduced ejection fraction: Real-world experience from Taiwan. Journal of Cardiovascular Pharmacology and Therapeutics.

[33] Ibrahim NE, McCarthy CP, Shrestha S, Gaggin HK, Mukai R, Szymonifka J, Apple FS, Burnett JC, Iyer S, Januzzi JL (2019). Effect of neprilysin inhibition on various natriuretic peptide assays. Journal of the American College of Cardiology.

[34] Sbolli M, deFilippi C (2020). BNP and NT-proBNP Interpretation in the neprilysin inhibitor era. Current Cardiology Reports.

[35] Ito S, Satoh M, Tamaki Y, Gotou H, Charney A, Okino N, Akahori M, Zhang J (2015) Safety and efficacy of LCZ696, a first-in-class angiotensin receptor neprilysin inhibitor, in Japanese patients with hypertension and renal dysfunction. *Hypertension Research, 38*(4):269–75. 10.1038/hr.2015.1. Epub 2015 Feb 19. PMID: 25693859; PMCID: PMC4396400. 10.1038/hr.2015.1 PMC4396400 25693859

[36] Imamura T, Hori M, Kinugawa K (2021) Optimal therapeutic strategy using sacubitril/valsartan in a patient with systolic heart failure and chronic kidney disease - An initial case report in Japan. *Internal Medicine, 60*(17):2807–2809. 10.2169/internalmedicine.6713-20. Epub 2021 Sep 1. PMID: 34470986; PMCID: PMC8479222. 10.2169/internalmedicine.6713-20 PMC8479222 34470986

[37] Nakamura M, Imamura T, Joho S, Kinugawa K (2021). Initial real-world practical experience of sacubitril/valsartan treatment in Japanese patients with chronic heart failure. Circulation Reports.

[38] Zeymer U, Clark AL, Barrios V, Damy T, Drożdż J, Fonseca C, Lund LH, Comite GD, Hupfer S, Maggioni AP (2020) Management of heart failure with reduced ejection fraction in Europe: Design of the ARIADNE registry. *ESC Heart Failure, 7*(2):727–736. 10.1002/ehf2.12569. Epub 2020 Feb 6. PMID: 32027782; PMCID: PMC7160498. 10.1002/ehf2.12569 PMC7160498 32027782

[39] Januzzi JL, Camargo CA, Anwaruddin S, Baggish AL, Chen AA, Krauser DG, Tung R, Cameron R, Nagurney JT, Chae CU, Lloyd-Jones DM, Brown DF, Foran-Melanson S, Sluss PM, Lee-Lewandrowski E, Lewandrowski KB (2005). The N-terminal Pro-BNP investigation of dyspnea in the emergency department (PRIDE) study. American Journal of Cardiology.

[40] Mueller C, McDonald K, de Boer RA, Maisel A, Cleland JGF, Kozhuharov N, Coats AJS, Metra M, Mebazaa A, Ruschitzka F, Lainscak M, Filippatos G, Seferovic PM, Meijers WC, Bayes-Genis A, Mueller T, Richards M, Januzzi JL Jr (2019) Heart Failure Association of the European Society of Cardiology. Heart Failure Association of the European Society of Cardiology practical guidance on the use of natriuretic peptide concentrations. *European Journal of Heart Failure, 21*(6):715–731. 10.1002/ejhf.1494. PMID: 31222929. 10.1002/ejhf.1494 31222929

[41] Myhre PL, Vaduganathan M, Claggett B, Packer M, Desai AS, Rouleau JL, Zile MR, Swedberg K, Lefkowitz M, Shi V, McMurray JJV, Solomon SD (2019). B-type natriuretic peptide during treatment with sacubitril/valsartan: The PARADIGM-HF Trial. Journal of the American College of Cardiology.

[42] Lee S, Oh J, Kim H, Ha J, Chun KH, Lee CJ, Park S, Lee SH, Kang SM (2020) Sacubitril/valsartan in patients with heart failure with reduced ejection fraction with end-stage of renal disease. *ESC Heart Failure*, *7*(3):1125–1129. 10.1002/ehf2.12659. Epub 2020 Mar 10. PMID: 32153122; PMCID: PMC7261577. 10.1002/ehf2.12659 PMC7261577 32153122

[43] Zhang H, Huang T, Shen W, Xu X, Yang P, Zhu D, Fang H, Wan H, Wu T, Wu Y, Wu Q (2020) Efficacy and safety of sacubitril-valsartan in heart failure: a meta-analysis of randomized controlled trials. *ESC Heart Failure*, *7*(6):3841–50. 10.1002/ehf2.12974. Epub ahead of print. PMID: 32977362; PMCID: PMC7754944. 10.1002/ehf2.12974 PMC7754944 32977362

[44] Damman K, Gori M, Claggett B, Jhund PS, Senni M, Lefkowitz MP, Prescott MF, Shi VC, Rouleau JL, Swedberg K, Zile MR, Packer M, Desai AS, Solomon SD, McMurray JJV (2018). Renal effects and associated outcomes during angiotensin-neprilysin inhibition in heart failure. JACC Heart Failure.

[45] Cho IJ, Kang SM (2021) Angiotensin receptor-neprilysin inhibitor in patients with heart failure and chronic kidney disease. *Kidney Research and Clinical Practice,**40*(4):555–565. 10.23876/j.krcp.21.900. Epub 2021 Nov 22. PMID: 34922429; PMCID: PMC8685363. 10.23876/j.krcp.21.900 PMC8685363 34922429

